# Effect of Melatonin on the Extracellular-Regulated Kinase Signal Pathway Activation and Human Osteoblastic Cell Line hFOB 1.19 Proliferation

**DOI:** 10.3390/ijms160510337

**Published:** 2015-05-07

**Authors:** Xiao-Chuan Xiong, Yue Zhu, Rui Ge, Li-Feng Liu, Wei Yuan

**Affiliations:** 1 Department of Orthopaedics, First Hospital, China Medical University, Shenyang 110001, China; E-Mails: doctxc@126.com (X.-C.X.); royvi218@sina.com (W.Y.); 2 Department of Orthopaedics, First Hospital, Dalian Medical University, Dalian 116011, China; E-Mail: cmu0204@126.com; 3 Department of Orthopaedics, East Hospital, Tongji University School of Medicine, Shanghai 200120, China; E-Mail: liulifengxy@163.com

**Keywords:** melatonin, osteoblasts, proliferation, extracellular signal-regulated kinase, signal transduction

## Abstract

It has been shown that melatonin may affect bone metabolism. However, it is controversial whether melatonin could promote osteoblast proliferation, and the precise molecular mechanism of melatonin on osteoblast proliferation is still obscure. In this study, the results of the CCK-8 assay showed that melatonin significantly promoted human osteoblastic cell line hFOB 1.19 cell proliferation at 1, 2.5, 5, 10, 25, 50 and 100 µM concentrations for 24 h, but there were no significant differences among the groups. Western blot demonstrated that 10 µM melatonin significantly promoted ERK1/2 phosphorylation. Furthermore, we also detected the phosphorylation of c-Raf, MEK1/2, p90RSK and MSK1, and all of them increased with 10 µM melatonin. U0126 (a selective inhibitor of MEK that disrupts downstream activation of ERK1/2) downregulated the phosphorylation of ERK1/2, p90RSK and MSK1. U0126 also attenuated the proliferation of osteoblasts stimulated by melatonin. In conclusion, this study for the first time indicates that melatonin (10 nM–100 µM) promotes the proliferation of a human osteoblastic cell line hFOB 1.19 through activation of c-Raf, MEK1/2, ERK1/2, p90RSK and MSK1.

## 1. Introduction

Melatonin is involved in many physiological processes [1,2,3,4], including sleep, anti-inflammatory functions, gastrointestinal physiology, regulation of body core temperature, blood pressure, cardiovascular function, immune regulatory properties, antioxidative defense, detoxification, reproduction, inhibition of apoptosis and bone physiology [3,4,5,6,7,8,9,10]. Some other organs also can synthesize melatonin, such as gastrointestinal tract, retina and bone marrow [1]. Mouse and human bone marrow cells are capable of *de novo* synthesis of melatonin, which may have intracellular and or paracrine functions [11,12]. In addition, rat bone marrow melatonin concentration is roughly two orders of magnitude higher than that in peripheral blood and displays a good correlation with circulating melatonin levels [13]. Accumulating evidence [14,15,16,17,18,19] from *in vitro* and *in vivo* experiments suggests that melatonin may affect bone metabolism. Some studies indicated that melatonin tended to promote osteoblasts differentiation and bone formation [14,15,16]. Park, K.H. *et al.* [20] indicated that melatonin promoted mouse osteoblastic MC3T3 cell differentiation. Radio, N.M. and Sethi, S. *et al.* [21,22] indicated that melatonin enhanced human adult mesenchymal stem cell (hAMSC) differentiation into osteoblasts. In the field of cell proliferation, Liu, L. *et al.* [12,23] indicated that melatonin has dual effects on osteoblast proliferation with different concentrations. Nakade, O. *et al.* [15] indicated that melatonin significantly and dose-dependently increased osteoblast proliferation, but Radio, N.M. and Roth, J.A. *et al.* [14,21] indicated that melatonin suppressed osteoblast proliferation. Thus, it can be seen that the study of melatonin on osteoblast proliferation is controversial.

The mechanisms of melatonin’s action on cells have been described as follows: (1) binding to intracellular proteins, such as calmodulin; (2) binding to nuclear receptors of the orphan family; and (3) binding to plasma membrane-localized melatonin receptors [24,25]. Most of the research has focused on melatonin plasma membrane receptors. Two distinct classes of melatonin plasma membrane receptors, which are expressed in humans, have been reported so far, MT1 and MT2 [24,26]. Melatonin plasma membrane receptors are a distinct group within the G protein-coupled receptor superfamily [24,26].

Previous studies indicated that melatonin influenced cAMP formation [27], protein kinase A activity and phosphorylation of the cAMP-responsive element binding protein (CREB) [26]. Melatonin also can stimulate c-Jun *N*-terminal kinase activity, influence the generation of intracellular Ca^2+^ [28] and promote PKC activity [29].

The ERK (extracellular-regulated kinases) signaling pathway can be activated in response to a diverse range of extracellular stimuli, including mitogens, growth factors, cytokines and hormones [30,31,32]. c-Raf, MEK1/2 (upstream of targets ERK1/2) and ERK1/2 can be activated in sequence when cells are stimulated by some active substances [32,33,34]. The activated ERK1/2 can activate the p90RSK (90-kDa ribosomal S6 kinase) and MSK1 (mitogen and stress-activated protein kinase) [35,36]. The Raf/MEK/ERK/p90RSK/MSK1 cascade couples signals can regulate gene expression and activity of many proteins involved in proliferation, apoptosis or differentiation [37]. Several studies demonstrated that melatonin promoted osteoblasts differentiation by activation of ERK1/2. Satué, M. *et al.* [38] indicated that melatonin promoted osteoblast differentiation via activation of ERK1/2. Park, K.H. *et al.* [20] indicated that melatonin promoted mouse osteoblastic MC3T3-E1 cell differentiation via the BMP/ERK/Wnt pathways. Radio, N.M. *et al.* [21] indicated that melatonin enhanced hAMSCs differentiation into osteoblasts via MT2 and the MEK/ERK (1/2) signaling cascade. Sethi, S. *et al.* [22] indicated that melatonin induced MT2/β-arrestin scaffolds complexed to Gi, MEK1/2 and ERK1/2 to localize ERK1/2 primarily in the cytosol, thus inducing hAMSCs differentiation into osteoblasts and affecting osteogenic gene expression.

However, the study of melatonin on osteoblast proliferation is controversial, and the precise molecular mechanisms of melatonin signaling pathways are still poorly understood. This study was performed to investigate the underlying mechanism of melatonin regarding how melatonin promotes osteoblast proliferation by activation of up- and down-stream targets of ERK1/2. This information can be helpful for exploring the signal transduction of melatonin in osteoblasts.

## 2. Results

### 2.1. Proliferative Effects of Melatonin on the Human Osteoblastic Cell Line hFOB 1.19

**Figure 1:**
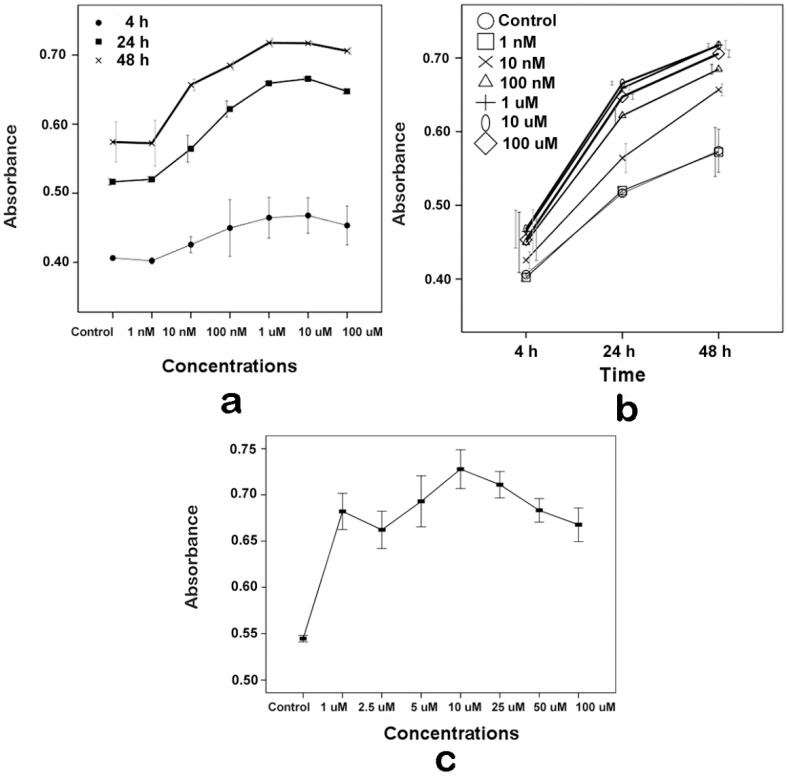
Effects of melatonin on hFOB 1.19 cell proliferation. Cell proliferation was measured by the CCK-8 assay. (**a**) The cells were exposed to various concentrations of melatonin for 4, 24 or 48 h. Results are represented as the dose-response effect curve; (**b**) The cells were exposed to various concentrations of melatonin for 4, 24 or 48 h. Results are represented as the time-response effect curve; and (**c**) The cells were exposed to 0 (Control), 1, 2.5, 5, 10, 25, 50 and 100 µM melatonin for 24 h. Results are represented as the dose-response effect curve. The results are represented as the means ± SEM of three independent experiments.

HFOB 1.19 cells are a normal human osteoblastic cell line [39,40]. To determine the effect of melatonin on the proliferation of hFOB 1.19 and to ascertain the specific concentration for the next step of research, the CCK-8 assay was performed on the hFOB cells. As shown in Figure 1a,b, melatonin’s effect was associated with concentrations and time respectively on the proliferation of hFOB 1.19, but the effect was not associated with the interaction of both variables (two-way ANOVA: concentrations, *p* = 0.000; time, *p* = 0.000; interaction, *p* = 0.144). Melatonin slightly promoted hFOB cell proliferation at indicated concentrations for 4 h, but there was no statistical significance compared with the control group. Melatonin significantly promoted hFOB cell proliferation at 10 nM–100 µM concentrations, in comparison with control cells at 24 and 48 h.

Furthermore, cells were exposed to 0 (Control), 1, 2.5, 5, 10, 25, 50 and 100 µM melatonin for 24 h, and the CCK-8 assay was also used to detect the effect of melatonin on hFOB cell proliferation. As shown in Figure 1c, melatonin significantly promoted hFOB cell proliferation at 1–100 µM concentrations compared with the control group. In addition, the results of the CCK-8 assay also showed that, at the 1–100 µM concentration range, there were no significant differences between different concentrations of each group.

### 2.2. Effects of Melatonin on the Human Osteoblastic Cell Line hFOB 1.19’s Viability

The trypan blue dye exclusion assay was used by optical counting of unstained viable cells in a hemocytometer to investigate cell viability. There was no obvious cells death when cells were exposed to 10 µM melatonin at any time point examined (cell viability, 4 h: 99% ± 0.1%; 24 h: 99% ± 0.3%; 48 h: 98% ± 0.5%).

### 2.3. Effects of Melatonin on the Expression of ERK in Human Osteoblastic Cell Line hFOB 1.19

**Figure 2:**
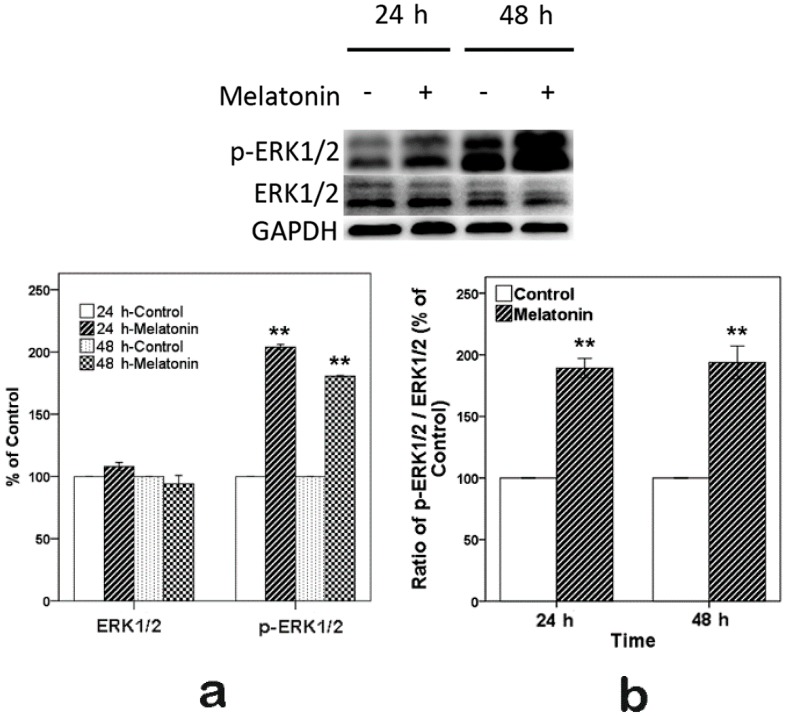
Expression of p-ERK1/2 induced by 10 µM melatonin in hFOB 1.19 cells for 24 and 48 h. (**a**) Total (ERK1/2) and phosphorylated (p-ERK1/2) protein expression levels. Expression levels were normalized by GAPDH; (**b**) Ratio of p-ERK1/2/ERK1/2. Expression levels were normalized by GAPDH first. Each bar is indicated as the relative percentage of control cells at 24 and 48 h. The results are represented as the means ± SEM of three independent experiments. ** *p* < 0.01, compared with the control cells.

To determine whether melatonin promoted osteoblast proliferation through the activation of ERK1/2, Western blot was used. The cells were exposed to 10 µM melatonin for 24 and 48 h, as shown in Figure 2. We observed that the expression of p-ERK1/2 (phosphorylation of ERK1/2) and the ratio of p-ERK1/2/ERK1/2 significantly increased in comparison with the control. Meanwhile, total ERK1/2 levels had no obvious change.

### 2.4. Effects of Melatonin and U0126 on the Expression of ERK in Human Osteoblastic Cell Line hFOB 1.19

**Figure 3:**
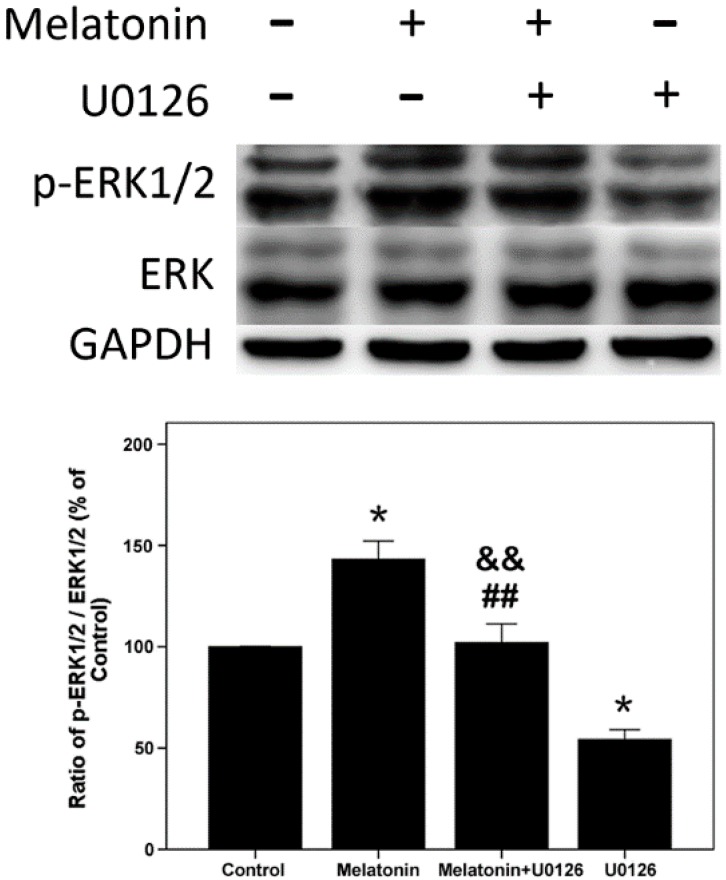
Expression of p-ERK1/2 induced by U0126 (20 µM), melatonin (10 µM) alone and both of them in hFOB 1.19 cells for 24 h. Total and phosphorylated (p-) expression levels were measured by Western blot. The results are represented as the ratio of p-ERK1/2/ERK1/2. Expression levels were normalized by GAPDH first. Each bar is indicated as the relative percentage of control cells. The results are represented as the means ± SEM of three independent experiments. * *p* < 0.05, compared with control cells; ^##^ *p* < 0.01, compared with the melatonin treatment group; ^&&^ *p* < 0.01, compared with the U0126 treatment group.

Furthermore, cells were exposed to U0126 (20 µM; MEK inhibitor, can inhibit downstream activation of ERK1/2), melatonin (10 µM) alone and both U0126 and melatonin for 24 h, as shown in Figure 3. Melatonin increased the ratio of p-ERK1/2/ERK1/2, and U0126 decreased the ratio of p-ERK1/2/ERK1/2. When cells were exposed to both melatonin and U0126, the ratio of p-ERK1/2/ERK1/2 was reduced compared with the melatonin treatment group, but increased compared with the U0126 treatment group.

### 2.5. Proliferative Effects of Melatonin and U0126 on the Human Osteoblastic Cell Line hFOB 1.19

**Figure 4:**
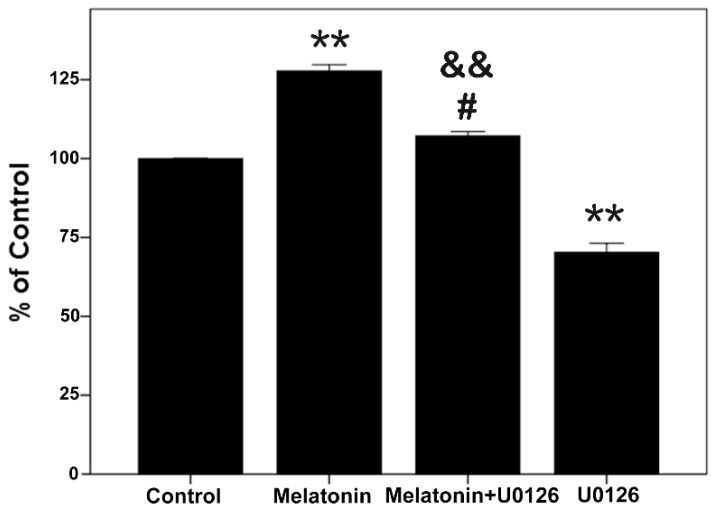
Effects of melatonin and U0126 on cell proliferation. The cells were exposed to U0126 (20 µM), melatonin (10 µM) alone and both of them for 24 h. Cell proliferation was measured by the CCK-8 assay. Results are indicated as the relative percentage of control cells. The results are represented as the means ± SEM of three independent experiments. ** *p* < 0.01, compared with control cells; ^#^ *p* < 0.05, compared with the melatonin treatment group; ^&&^ *p* < 0.01, compared with the U0126 treatment group.

Meanwhile, the CCK-8 assay results, as shown in Figure 4, showed simultaneously that melatonin significantly promoted proliferation and that U0126 inhibited proliferation. When cells were exposed to both melatonin and U0126, the proliferation effect of melatonin was attenuated compared with the melatonin treatment group, but promoted proliferation compared with the U0126 treatment group. All results above indicate that 10 µM melatonin induced osteoblast proliferation is associated with activation of ERK1/2.

### 2.6. Effects of Melatonin on the Expressions of Raf, MEK, p90RSK and MSK1 in Human Osteoblastic Cell Line hFOB 1.19

**Figure 5:**
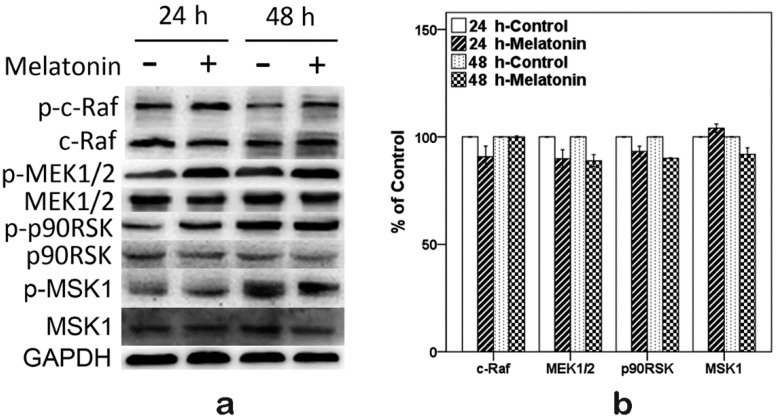

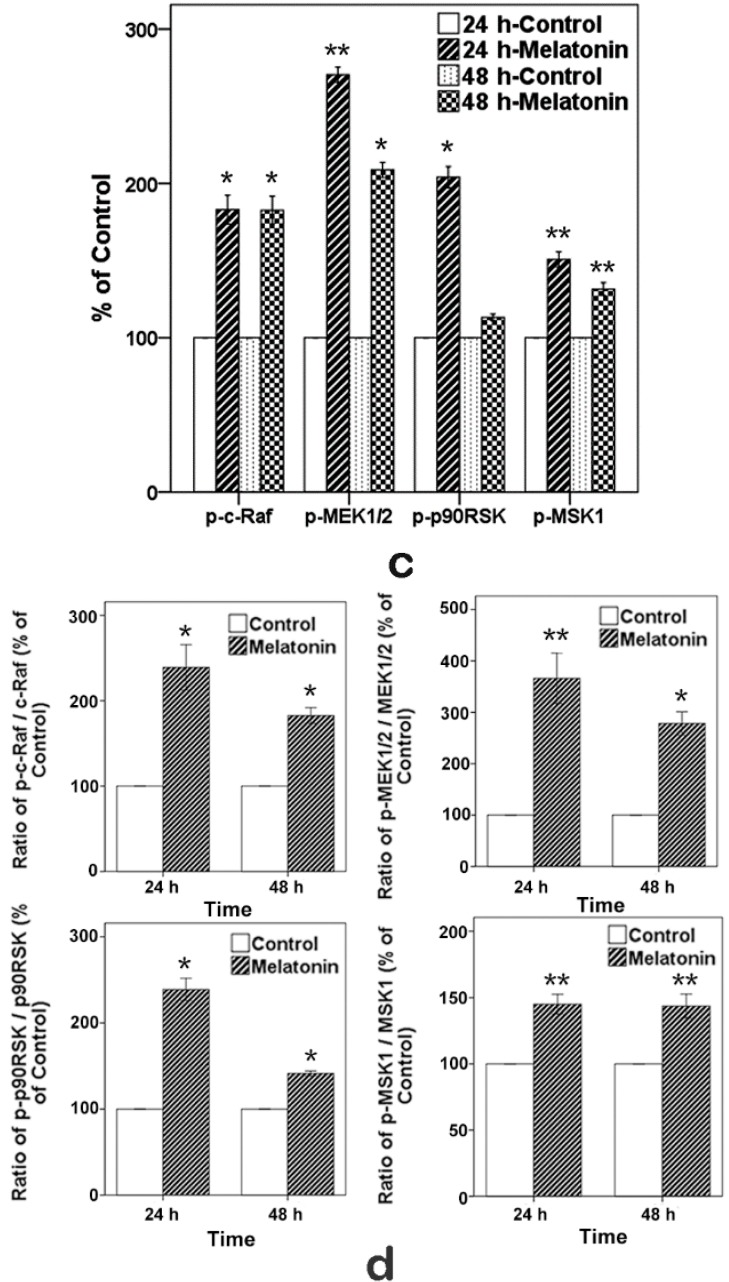
Expressions of p-c-Raf, p-MEK1/2, p-p90RSK and p-MSK1 induced by 10 µM melatonin in hFOB 1.19 cells for 24 and 48 h. (**a**) Total and phosphorylated (p-) expression levels were measured by Western blot; (**b**) Total protein expression levels. Expression levels were normalized by GAPDH; (**c**) Phosphorylated (p-) protein expression levels. Expression levels were normalized by GAPDH; and (**d**) Ratio of phosphorylated protein/total protein expression levels. Expression levels were normalized by GAPDH first. Each bar is indicated as the relative percentage of control cells. The results are represented as the means ± SEM of three independent experiments. * *p* < 0.05 or ** *p* < 0.01, compared with control cells.

In order to clarify how melatonin induced the expression of p-ERK1/2 and how melatonin promoted osteoblast proliferation, Western blot was performed to detect the p-c-Raf, p-MEK1/2, p-p90RSK and p-MSK1. As shown in Figure 5, the expressions of p-c-Raf, p-MEK1/2, p-p90RSK, p-MSK1 and the ratio of phosphorylated protein/total protein were increased when cells were exposed to 10 µM melatonin for 24 and 48 h. Meanwhile, total protein (c-Raf, MEK1/2, p90RSK and MSK1) levels had no obvious change.

### 2.7. Effects of Melatonin and U0126 on the Expressions of p90RSK and MSK1 in Human Osteoblastic Cell Line hFOB 1.19

**Figure 6:**
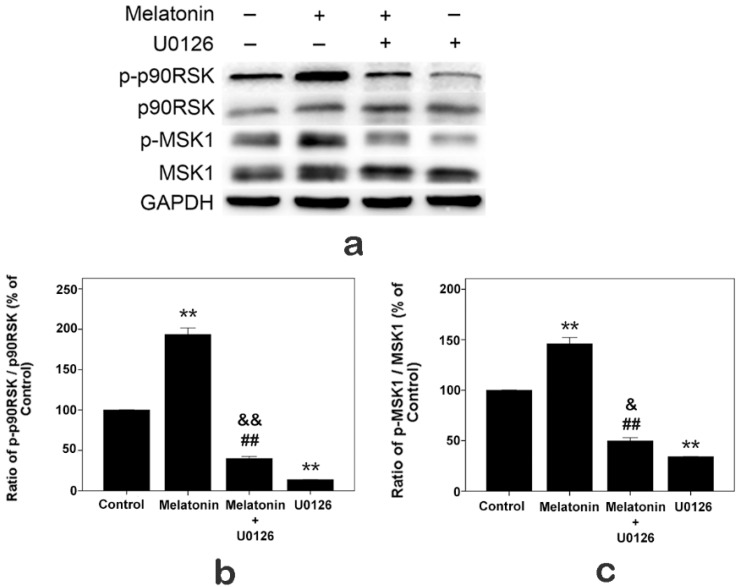
Expressions of p-p90RSK and p-MSK1 induced by U0126 (20 µM), melatonin (10 µM) alone and both U0126 and melatonin in hFOB 1.19 cells for 24 h. (**a**) Total and phosphorylated (p-) expression levels were measured by Western blot; (**b**) ratio of p-p90RSK/p90RSK; (**c**) ratio of p-MSK1/MSK1. Expression levels were normalized by GAPDH first. Each bar is indicated as the relative percentage of control cells. The results are represented as the means ± SEM of three independent experiments. ** *p* < 0.01, compared with control cells; ^##^ *p* < 0.01, compared with the melatonin treatment group; ^&&^ *p* < 0.01, ^&^ *p* < 0.05, compared with the U0126 treatment group.

Furthermore, cells were exposed to U0126 (20 µM), melatonin (10 µM) alone and both U0126 and melatonin for 24 h. As shown in Figure 6, U0126 inhibited the ratio of p-p90RSK/p90RSK and p-MSK1/MSK1, and when cells were exposed to both melatonin and U0126, the ratios of p-p90RSK/p90RSK and p-MSK1/MSK1 were reduced compared with the melatonin treatment group, but increased compared with the U0126 treatment group.

## 3. Discussion

Melatonin regulates a variety of physiological and pathophysiological processes [1,2,3,4]. It has been shown to influence cell proliferation and differentiation [14,15,16,41]. In this study, the results of the CCK-8 assay demonstrated that melatonin (10 nM–100 µM) promoted osteoblast proliferation. The results are consistent with previous research [12,15]. To determine the effect of melatonin on the proliferation of hFOB 1.19 and to ascertain the specific concentration for the next step of research, the results of the CCK-8 assay also demonstrated that melatonin significantly promoted proliferation at 1–100 µM, but there were no significant differences among the groups of 1, 2.5, 5, 10, 25, 50 and 100 µM concentrations.

Several studies demonstrated that melatonin promoted osteoblast differentiation. However, only a few studies focused on osteoblast proliferation, and the results were controversial [12,14,15]. Our results indicated that melatonin promoted osteoblast proliferation at 10 nM–100 µM concentrations.

According to the results of the CCK-8 assay, we chose 10 µM melatonin for the next step of the research. Activation of ERK1/2 has been well proven to be necessary for cell proliferation [30,42]. Previous studies [43,44,45] using other cell types indicated that melatonin increased p-ERK1/2. Therefore, we sought to determine whether p-ERK1/2 was increased when the hFOB cells were exposed to 10 µM melatonin. The Western blot results suggested that 10 µM melatonin increased p-ERK1/2, which is consistent with previous reports [20,38]. 

The ERK signal pathway regulates the cell proliferation, differentiation and survival [46,47,48]. The p-ERK1/2 is just an intermediate in signal pathways, and many upstream and downstream targets of ERK1/2 have been identified [32,33,34,35,49]. Although several studies demonstrated that melatonin affects the activation of ERK1/2 [20,23,38], only a few studies are available regarding up- and down-stream targets of activated ERK1/2 when melatonin promotes osteoblast proliferation. The Raf/MEK/ERK/p90RSK/MSK1 cascade couples signals can transmit information into the nucleus, thus regulating gene expression. This pathway has diverse effects, which can regulate cell proliferation, apoptosis or differentiation [37]. In this study, after we confirmed that melatonin promoted the hFOB cell proliferation and increased the expression of p-ERK1/2, we sought to determine whether the Raf/MEK/ERK/p90RSK/MSK1 signal pathway was activated when melatonin promoted proliferation of the hFOB cells. We detected the activation of c-Raf, MEK1/2, p90RSK and MSK1. Our results suggested that 10 µM melatonin significantly increased the phosphorylation of c-Raf, MEK1/2, p90RSK and MSK1. Meanwhile, total protein (c-Raf, MEK1/2, ERK1/2, p90RSK and MSK1) levels had no obvious change. In addition, U0126 (a selective inhibitor of MEK, which can inhibit downstream activation of ERK1/2) attenuated the proliferation effect of melatonin through inhibiting the activation of ERK1/2 and its downstream p90RSK, MSK1. These results indicate that 10 µM melatonin significantly promotes osteoblast proliferation via activation of the c-Raf/MEK/ERK/p90RSK/MSK1 signal pathway.

Melatonin is considered as an effective drug for many diseases, such as insomnia, cancer and osteoporosis. A randomized, double-blind, placebo-controlled study showed that melatonin supplementation restored imbalances in bone remodeling to prevent bone loss [19]. However, the precise molecular mechanisms of the melatonin signal pathway are still unclear. In the field of cell differentiation, Park, K.H. *et al.* [20] indicated that melatonin (50 nM for 5 days) promoted mouse osteoblastic MC3T3 cell differentiation. Satué, M. *et al.* [38] indicated that melatonin (0.1 mM melatonin for 2 h after being cultured for seven days) promoted MC3T3 (mouse pre-osteoblasts) differentiation via activation of ERK1/2. Sethi, S. *et al.* [22] indicated that melatonin (50 nM melatonin for 2, 5, 10, 14 and 21 days) induced MT_2_/β-arrestin scaffolds complexed to Gi, MEK1/2 and ERK1/2 to localize ERK1/2 primarily in cytosol, thus inducing human adult mesenchymal stem cell (hAMSC) differentiation into osteoblasts and affecting osteogenic gene expression. Bondi, C.D. *et al.* [50] found the same mechanism in Chinese hamster ovary cells. Radio, N.M. *et al.* [21] indicated that melatonin (50 nM melatonin for 10 days) enhanced hAMSC differentiation into osteoblasts via MT2 melatonin receptors and the ERK1/2 signaling cascade. In the field of cell proliferation, Liu, L. *et al.* [12] indicated that melatonin (1 nM–100 µM melatonin promotion and 1 mM melatonin inhibition for 1, 2 and 3 days) had dual effects on human fetal osteoblastic cell line hFOB 1.19 proliferation with different concentrations. Sethi, S. *et al.* [22] indicated that 50 nM melatonin slightly promoted hAMSC proliferation for two days, but slightly inhibited hAMSC proliferation for 10 and 21 days. Nakade, O. *et al.* [15] indicated that melatonin significantly and dose-dependently increased human osteoblast (HOB-M cells and SV-HFO cells, 5–100 µM melatonin for one day) proliferation, but Liu, L. *et al.* [51] indicated that melatonin (4–10 mM melatonin for 1, 2 and 3 days) inhibited the proliferation of human osteosarcoma cell line MG-63. Roth, J.A. *et al.* [14] indicated that melatonin (50 nM melatonin for one day) slightly suppressed MC3T3 cell and ROS (rat osteoblast-like osteosarcoma 17/2.8 cell) cell proliferation when melatonin promoted differentiation. Radio, N.M. *et al.* [21] also indicated that 50 nM melatonin inhibited the proliferation of hAMSCs for seven days. Our results indicated that melatonin promoted human fetal osteoblastic cell line hFOB 1.19 proliferation at 10 nM–100 µM concentrations via the ERK1/2 signaling cascade. The data available indicate that melatonin has different cell effects depending on the concentrations and duration of melatonin or the cell type examined. What are the causes of this phenomenon?

Luttrell, L.M. *et al.* [52] and DeFea, K.A. *et al.* [53] indicated that the complex of the internalized receptor, β-arrestin and activated ERK1/2, is required for ERK1/2 activation. ERK1/2 activity is retained in the cytosol and neither translocates to the nucleus nor causes proliferation. However, activated ERK1/2 will promote cell proliferation when cells do not form the complex, and the activated ERK1/2 is translocated to the nucleus. Radio, N.M. and Sethi, S. *et al.* [21,22] indicated that melatonin induced the formation of the complex to localize the activated ERK1/2 primarily in cytosol when melatonin promoted hAMSC differentiation into osteoblasts and inhibited the proliferation of hAMSCs. Our results indicated that melatonin promoted human fetal osteoblastic cell line hFOB 1.19 proliferation via activating of ERK1/2. When melatonin activated ERK1/2, different cells effects were induced by melatonin. Therefore, we speculate that the possible cause of melatonin’s different cell effects might be associated with the localization of activated ERK1/2 in cells. Different concentrations and durations of melatonin or the cell type examined may induce different localization of activated ERK1/2 in cells, thus producing different cell effects.

The way of melatonin acts on cells is diverse. Research showed that human osteoblasts have plasma membrane melatonin receptors [21,22]. However, so far, Mechanisms (1) and (2) of melatonin (see the Introduction) in osteoblasts are still unclear. Another cause of melatonin’s different cell effects might be that different cell types exposed to different concentrations and durations of melatonin may induce different actions of melatonin on cells. In addition, some other organs also can synthesize melatonin; mouse and human bone marrow cells are capable of *de novo* synthesis of melatonin, which may have intracellular and or paracrine functions [11]. Bone marrow cells cultured for a prolonged period exhibited high levels of melatonin [13]. It really should be noted that local high melatonin levels may have more impact on cells than circulating melatonin. When cells are exposed to exogenous melatonin, the endogenous melatonin produced by the cells themselves may influence the effects of exogenous melatonin. Endogenous melatonin and different actions of melatonin on cells may lead to the cells losing the dose-response effect to exogenous melatonin. Thus, melatonin may either stimulate or suppress cell proliferation depending on the concentrations and duration of melatonin or the cell type examined. This feature limits the application of melatonin in disease treatment. However, it is necessary to clarify the effect and the signal transduction of melatonin for various kinds of cells and whether these cells synthesize melatonin.

This study clearly indicates that melatonin significantly promotes osteoblast proliferation via activation of the c-Raf/MEK/ERK/p90RSK/MSK1 signal pathway. The results of the trypan blue dye exclusion assay indicated that melatonin has almost no cytotoxicity to osteoblasts, which is consistent with a previous study [38]. The study findings may provide evidence for melatonin in the treatment of osteoporosis. However, it is worth noting that melatonin can either stimulate or suppress osteoblast proliferation depending on the concentrations and duration of melatonin treatment [12,14,15]. The application of melatonin for the treatment of osteoporosis should be done with caution, and more clinical trials are needed.

In conclusion, this study for the first time indicates that melatonin (10 nM–100 µM) promotes the proliferation of human osteoblastic cell line hFOB 1.19 through activation of c-Raf, MEK1/2, ERK1/2, p90RSK and MSK1. The study findings assist with the clarification of the signal transduction of melatonin on human osteoblasts and provides evidence for melatonin for the treatment of osteoporosis.

## 4. Materials and Methods

### 4.1. Materials

Primary monoclonal antibodies for ERK1/2, phosphorylation of ERK1/2 (p-ERK1/2), c-Raf, phosphorylation of Raf (p-c-Raf), MEK1/2, phosphorylation of MEK1/2 (p-MEK1/2), p90RSK, phosphorylation of 90RSK (p-p90RSK), MSK1, phosphorylation of MSK1 (p-MSK1), GAPDH and U0126 (MEK Inhibitor) were purchased from Cell Signaling Technology (Danvers, MA, USA).

### 4.2. Cell Culture

The cell line hFOB 1.19 (human fetal osteoblastic cell line [39]), kindly provided by Malayannan Subramaniam [40], was cultured in a 1:1 mixture of Ham’s F12 Medium Dulbecco’s Modified Eagle’s Medium, without phenol red (Hyclone, Thermo, Fremont, CA, USA). To make the complete growth medium, we added the G418 (0.3 mg/mL) to the base medium and supplement with 10% fetal bovine serum (FBS) (Hyclone, Thermo) in a humidified 5% CO_2_ atmosphere at 37 °C. The medium was changed every other day. The hFOB cells were utilized in Passages 10–15 and plated at 10^4^ cells/cm^2^ for 24 h before treatment. Cells were treated with melatonin, which was dissolved in 0.2% dimethyl sulfoxide (DMSO) or vehicle (0.2% DMSO in culture medium only) media containing 10% FBS.

### 4.3. Cell Proliferation Assay

According to the manufacturer’s instructions, osteoblast proliferative activity was assessed using the Cell Counting Kit-8 (CCK-8; Dojindo, Kumamoto, Japan). Briefly, hFOB 1.19 cells were seeded in 96-well plates for 24 h, and then, the cells were exposed to melatonin of the indicated concentrations for 4, 24 and 48 h (Figure 1a,b), or exposed to 0, 1, 2.5, 5, 10, 25, 50 and 100 µM melatonin for 24 h (Figure 1c), or exposed to 10 µM melatonin, 20 µM U0126 and both 20 µM U0126 and 10 µM melatonin for 24 h (Figure 4). Then, the medium was discarded and replaced with 100 μL of fresh medium containing 10% CCK-8, and the plates were incubated at 37 °C for 4 h; the absorbance was detected at 450 nm with a microplate reader.

### 4.4. Trypan Blue Dye Exclusion Assay

After treatment with 10 µM melatonin, the cells were collected by trypsinization and suspended in culture medium. Viable and nonviable cells were then determined by direct counting using a hemocytometer in the presence of 0.4% trypan blue. Cell viability was then assessed as the percentage of viable cells relative to the total population.

### 4.5. Western Blotting

The cells were extracted with lysis buffer (150 mM NaCl, 1% NP-40, 0.1% SDS, 2 µg/mL aprotinin, 1 mM PMSF) for 30 min at 4 °C. We centrifuged the cell lysate at 12,000× *g* for 15 min at 4 °C. The supernatant fluid (total cell lysate) was harvested. We transferred the supernatant to a new microcentrifuge tube. Aliquots, each containing 50 µg of protein, were separated by 10% SDS-PAGE and transferred to PVDF membranes at 90 V for 70 min at a low temperature. The membranes were blocked in 5% bovine serum albumin (phospho-) or skim milk (the others) for 2 h. Proteins were detected using a 1:1000 (GAPDH) or 1:500 (the others) dilution overnight at 4 °C and then using anti-rabbit IgG conjugated with horse radish peroxidase at 1:8000 dilution for 2 h at room temperature, respectively. The EC3 Imaging System (UVP LLC, Upland, CA, USA) was used to scan the specific bands, and the optical density of each band was measured using Gel-Pro Analyzer 4.0 software (Media Cybernetics Inc., Bethesda, MD, USA). All of the bands were normalized by GAPDH. Then, phosphorylated (p-) proteins were normalized by total proteins (the ratio of phosphorylated proteins/total proteins).

### 4.6. Statistical Analysis

Data were analyzed using SPSS 22.0 statistical software (SPSS Inc., Chicago, IL, USA). Two-way ANOVA was used to assess whether differences were owed to melatonin concentrations, to the time of exposure or to the interaction of both variables. One-way ANOVA was used to evaluate the differences between groups with various treatments. The Tukey HSD test was used for post hoc subgroup analysis; an independent-sample *t*-test was used to evaluate the differences between groups with various treatments. All data are expressed as the mean ± SEM of at least three independent experiments. *p* < 0.05 was considered statistically significant.

## References

[1] Sánchez-Barceló E.J., Mediavilla M.D., Tan D.X., Reiter R.J. (2010). Scientific basis for the potential use of melatonin in bone diseases: Osteoporosis and adolescent idiopathic scoliosis. J. Osteoporos..

[2] Navarro-Alarcón M., Ruiz-Ojeda F.J., Blanca-Herrera R.M., A-Serrano M.M., Acuña-Castroviejo D., Fernández-Vázquez G., Agil A. (2014). Melatonin and metabolic regulation: A review. Food Funct..

[3] Cardinali D.P., Ladizesky M.G., Boggio V., Cutrera R.A., Mautalen C. (2003). Melatonin effects on bone: Experimental facts and clinical perspectives. J. Pineal Res..

[4] Witt-Enderby P.A., Radio N.M., Doctor J.S., Davis V.L. (2006). Therapeutic treatments potentially mediated by melatonin receptors: Potential clinical uses in the prevention of osteoporosis, cancer and as an adjuvant therapy. J. Pineal Res..

[5] Touitou Y. (2001). Human aging and melatonin. Clinical relevance. Exp. Gerontol..

[6] Naji L., Carrillo-Vico A., Guerrero J.M., Calvo J.R. (2004). Expression of membrane and nuclear melatonin receptors in mouse peripheral organs. Life Sci..

[7] Chucharoen P., Chetsawang B., Srikiatkhachorn A., Govitrapong P. (2003). Melatonin receptor expression in rat cerebral artery. Neurosci. Lett..

[8] Carrillo-Vico A., Garcia-Perganeda A., Naji L., Calvo J.R., Romero M.P., Guerrero J.M. (2003). Expression of membrane and nuclear melatonin receptor mRNA and protein in the mouse immune system. Cell. Mol. Life Sci..

[9] Delagrange P., Atkinson J., Boutin J.A., Casteilla L., Lesieur D., Misslin R., Pellissier S., Pénicaud L., Renard P. (2003). Therapeutic perspectives for melatonin agonists and antagonists. J. Neuroendocrinol..

[10] Zhao H., Poon A.M., Pang S.F. (2000). Pharmacological characterization, molecular subtyping, and autoradiographic localization of putative melatonin receptors in uterine endometrium of estrous rats. Life Sci..

[11] Conti A., Conconi S., Hertens E., Skwarlo-Sonta K., Markowska M., Maestroni J.M. (2000). Evidence for melatonin synthesis in mouse and human bone marrow cells. J. Pineal Res..

[12] Liu L., Zhu Y., Xu Y., Reiter R.J. (2011). Melatonin delays cell proliferation by inducing G_1_ and G_2_/M phase arrest in a human osteoblastic cell line hFOB 1.19. J. Pineal Res..

[13] Tan D.X., Manchester L.C., Reiter R.J., Qi W.B., Zhang M., Weintraub S.T., Cabrera J., Sainz R.M., Mayo J.C. (1999). Identification of highly elevated levels of melatonin in bone marrow: Its origin and significance. Biochim. Biophys. Acta.

[14] Roth J.A., Kim B.G., Lin W.L., Cho M.I. (1999). Melatonin promotes osteoblast differentiation and bone formation. J. Biol. Chem..

[15] Nakade O., Koyama H., Ariji H., Yajima A., Kaku T. (1999). Melatonin stimulates proliferation and type I collagen synthesis in human bone cells *in vitro*. J. Pineal Res..

[16] Satomura K., Tobiume S., Tokuyama R., Yamasaki Y., Kudoh K., Maeda E., Nagayama M. (2007). Melatonin at pharmacological doses enhances human osteoblastic differentiation *in vitro* and promotes mouse cortical bone formation *in vivo*. J. Pineal Res..

[17] Man G.C., Wang W.W., Yeung B.H., Lee S.K., Ng B.K., Hung W.Y., Wong J.H., Ng T.B., Qiu Y., Cheng J.C. (2010). Abnormal proliferation and differentiation of osteoblasts from girls with adolescent idiopathic scoliosis to melatonin. J. Pineal Res..

[18] Sanchez-Hidalgo M., Lu Z., Tan D.X., Maldonado M.D., Reiter R.J., Gregerman R.I. (2007). Melatonin inhibits fatty acid-induced triglyceride accumulation in ROS17/2.8 cells: Implications for osteoblast differentiation and osteoporosis. Am. J. Physiol. Regul. Integr. Comp. Physiol..

[19] Kotlarczyk M.P., Lassila H.C., O’Neil C.K., D’Amico F., Enderby L.T., Witt-Enderby P.A., Balk J.L. (2012). Melatonin osteoporosis prevention study (MOPS): A randomized, double-blind, placebo-controlled study examining the effects of melatonin on bone health and quality of life in perimenopausal women. J. Pineal Res..

[20] Park K.H., Kang J.W., Lee E.M., Kim J.S., Rhee Y.H., Kim M., Jeong S.J., Park Y.G., Kim S.H. (2011). Melatonin promotes osteoblastic differentiation through the BMP/ERK/Wnt signaling pathways. J. Pineal Res..

[21] Radio N.M., Doctor J.S., Witt-Enderby P.A. (2006). Melatonin enhances alkaline phosphatase activity in differentiating human adult mesenchymal stem cells grown in osteogenic medium via MT2 melatonin receptors and the MEK/ERK1/2 signaling cascade. J. Pineal Res..

[22] Sethi S., Radio N.M., Kotlarczyk M.P., Chen C.T., Wei Y.H., Jockers R., Witt-Enderby P.A. (2010). Determination of the minimal melatonin exposure required to induce osteoblast differentiation from human mesenchymal stem cells and these effects on downstream signaling pathways. J. Pineal Res..

[23] Liu L., Zhu Y., Xu Y., Reiter R.J. (2012). Prevention of ERK activation involves melatonin-induced G_1_ and G_2_/M phase arrest in the human osteoblastic cell line hFOB 1.19. J. Pineal Res..

[24] Ekmekcioglu C. (2006). Melatonin receptors in humans: Biological role and clinical relevance. Biomed. Pharmacother..

[25] Macchi M.M., Bruce J.N. (2004). Human pineal physiology and functional significance of melatonin. Front. Neuroendocrinol..

[26] Dubocovich M.L., Rivera-Bermudez M.A., Gerdin M.J., Masana M.I. (2003). Molecular pharmacology, regulation and function of mammalian melatonin receptors. Front. Biosci..

[27] Witt-Enderby P.A., Masana M.I., Dubocovich M.L. (1998). Physiological exposure to melatonin supersensitizes the cyclic adenosine 3',5'-monophosphate-dependent signal transduction cascade in Chinese hamster ovary cells expressing the human mt1 melatonin receptor. Endocrinology.

[28] Domínguez-Alonso A., Valdés-Tovar M., Solís-Chagoyán H., Benítez-King G. (2015). Melatonin stimulates dendrite formation and complexity in the hilar zone of the rat hippocampus: Participation of the Ca^++^/calmodulin complex. Int. J. Mol. Sci..

[29] Godson C., Reppert S.M. (1997). The Mel1a melatonin receptor is coupled to parallel signal transduction pathways. Endocrinology.

[30] Roux P.P., Blenis J. (2004). ERK and p38 MAPK-activated protein kinases: A family of protein kinases with diverse biological functions. Microbiol. Mol. Biol. Rev..

[31] Meloche S., Pouysségur J. (2007). The ERK1/2 mitogen-activated protein kinase pathway as a master regulator of the G_1_- to S-phase transition. Oncogene.

[32] Baccarini M. (2005). Second nature: Biological functions of the Raf-1 “kinase”. FEBS Lett..

[33] Murphy L.O., Blenis J. (2006). MAPK signal specificity: The right place at the right time. Trends Biochem. Sci..

[34] Rubinfeld H., Seger R. (2005). The ERK cascade: A prototype of MAPK signaling. Mol. Biotechnol..

[35] Dalby K.N., Morrice N., Caudwell F.B., Avruch J., Cohen P. (1998). Identification of regulatory phosphorylation sites in mitogen-activated protein kinase (MAPK)-activated protein kinase-1a/p90rsk that are inducible by MAPK. J. Biol. Chem..

[36] Deak M., Clifton A.D., Lucocq L.M., Alessi D.R. (1998). Mitogen- and stress-activated protein kinase-1 (MSK1) is directly activated by MAPK and SAPK2/p38, and may mediate activation of CREB. EMBO J..

[37] McCubrey J.A., Steelman L.S., Chappell W.H., Abrams S.L., Wong E.W., Chang F., Lehmann B., Terrian D.M., Milella M., Tafuri A. (2007). Roles of the Raf/MEK/ERK pathway in cell growth, malignant transformation and drug resistance. Biochim. Biophys. Acta.

[38] Satué M., Ramis J.M., del Mar Arriero M., Monjo M. (2015). A new role for 5-methoxytryptophol on bone cells function *in vitro*. J. Cell. Biochem..

[39] Harris S.A., Enger R.J., Riggs B.L., Spelsberg T.C. (1995). Development and characterization of a conditionally immortalized human fetal osteoblastic cell line. J. Bone Miner Res..

[40] Subramaniam M., Jalal S.M., Rickard D.J., Harris S.A., Bolander M.E., Spelsberg T.C. (2002). Further characterization of human fetal osteoblastic hFOB 1.19 and hFOB/ERα cells: Bone formation *in vivo* and karyotype analysis using multicolor fluorescent *in situ* hybridization. J. Cell. Biochem..

[41] Rodriguez C., Martín V., Herrera F., García-Santos G., Rodriguez-Blanco J., Casado-Zapico S., Sánchez-Sánchez A.M., Suárez S., Puente-Moncada N., Anítua M.J. (2013). Mechanisms involved in the pro-apoptotic effect of melatonin in cancer cells. Int. J. Mol. Sci..

[42] Chambard J.C., Lefloch R., Pouysse´gur J., Lenormand P. (2007). ERK implication in cell cycle regulation. Biochim. Biophys. Acta.

[43] Kilic U., Kilic E., Reiter R.J., Bassetti C.L., Hermann D.M. (2005). Signal transduction pathways involved in melatonin-induced neuroprotection after focal cerebral ischemia in mice. J. Pineal Res..

[44] Koh P.O. (2008). Melatonin attenuates the cerebral ischemic injury via the MEK/ERK/p90RSK/bad signaling cascade. J. Vet. Med. Sci..

[45] Luchetti F., Betti M., Canonico B., Arcangeletti M., Ferri P., Galli F., Papa S. (2009). ERK MAPK activation mediates the antiapoptotic signaling of melatonin in UVB-stressed U937 cells. Free Radic. Biol. Med..

[46] Bonni A., Brunet A., West A.E., Datta S.R., Takasu M.A., Greenberg M.E. (1999). Cell survival promoted by the Ras-MAPK signaling pathway by transcription-dependent and -independent mechanisms. Science.

[47] Pearson G., Robinson F., Beers Gibson T., Xu B.E., Karandikar M., Berman K., Cobb M.H. (2001). Mitogen-activated protein (MAP) kinase pathways: Regulation and physiological functions. Endocr. Rev..

[48] Shimamura A., Ballif B.A., Richards S.A., Blenis J. (2000). Rsk1 mediates a MEK-MAP kinase cell survival signal. Curr. Biol..

[49] Marais R., Wynne J., Treisman R. (1993). The SRF accessory protein Elk-1 contains a growth factor-regulated transcriptional activation domain. Cell.

[50] Bondi C.D., McKeon R.M., Bennett J.M., Ignatius P.F., Brydon L., Jockers R., Melan M.A., Witt-Enderby P.A. (2008). MT1 melatonin receptor internalization underlies melatonin-induced morphologic changes in Chinese hamster ovary cells and these processes are dependent on Gi proteins, MEK1/2 and microtubule modulation. J. Pineal Res..

[51] Liu L., Xu Y., Reiter R.J. (2013). Melatonin inhibits the proliferation of human osteosarcoma cell line MG-63. Bone.

[52] Luttrell L.M., Roudabush F.L., Choy E.W., Miller W.E., Field M.E., Pierce K.L., Lefkowitz R.J. (2001). Activation and targeting of extracellular signal-regulated kinases by β-arrestin scaffolds. Proc. Natl. Acad. Sci. USA.

[53] DeFea K.A., Zalevsky J., Thoma M.S., Déry O., Mullins R.D., Bunnett N.W. (2000). β-Arrestin-dependent endocytosis of proteinase-activated receptor 2 is required for intracellular targeting of activated ERK1/2. J. Cell Biol..

